# Supplementary material for: Comparison of Endoscopic Submucosal Dissection and Radical Surgery for Early Gastric Cancer in Remnant Stomach
Source: J Clin Med. 2022 Sep 14;11(18):5403. doi: 10.3390/jcm11185403 (PMC9503103; doi:10.3390/jcm11185403)
Supplement: Supplementary file 1 [file jcm-11-05403-s001.zip › jcm-1901400-supplementary.pdf]

Supplementary table S1 Clinical and pathological outcomes of previous lesion background

	ESD (n=48)	Surgery (n=25)
Histologic type of previous lesion		
Ulcer	1	6
NA	14	3
Mesenchymoma	1	1
Liomyoma	0	1
Differentiated	17	5
Undifferentiated	15	9
T staging		
Inapplicability (Ulcer)	1	6
NA	16	9
T1a	3	1
T1b	8	3
T2	5	2
T3	7	3
T4a	8	1
N staging	47	19
Inapplicability (Ulcer)		
NA	16	9
N0	20	6
N1	5	3
N2	2	0
N3a	2	1
N3b	2	0
TNM staging		
Inapplicability (Ulcer)	1	6
NA	16	9
IA	6	1
IB	5	3
IIA	8	4
IIB	3	1
IIIA	4	0
IIIB	3	1
IIIC	2	0
Additional treatment after previous surgery	33	14
Chemotherapy	25	12
Radiotherapy	8	2

NA, Not available

Supplementary table S2 Comparison of T staging in previous lesion background between the dead and the living

	Inapplicability (Ulcer)	NA	T1a	T1b	T2	T3	T4a	Total
Dead	2	6	0	1	0	0	3	12
Alive	5	19	4	10	7	10	6	61
Total	7	25	4	11	7	10	9	73

NA, Not available

Supplementary table S3 Comparison of N staging in previous lesion background between the dead and the living

	Inapplicability (Ulcer)	NA	N0	N1	N2	N3a	N3b	Total
Dead	2	6	1	0	1	0	2	12
Alive	5	19	25	8	1	3	0	61
Total	7	25	26	8	2	3	2	73

NA, Not available

Supplementary table S4 Comparison of TNM staging in previous lesion background between the dead and the living

	Inapplicability (Ulcer)	NA	IA	IB	IIA	IIB	IIIA	IIIB	IIIC	Total
Dead	2	6	0	1	0	0	1	0	2	12
Alive	5	19	7	7	12	4	3	4	0	61
Total	7	25	7	8	12	4	4	4	2	73

NA, Not available
